# Education and regular Feedback for Antimicrobial Stewardship in nursing homes: EduFAST study protocol

**DOI:** 10.3389/fmed.2026.1787035

**Published:** 2026-05-22

**Authors:** Irene Sotillo-Sánchez, Laura Herrera-Hidalgo, Cintia Flores-Rubio, Lydia Gálvez-Benítez, Eva María Herrera-Hernández, Germán Peñalva-Moreno, Marta Mejías-Trueba, Didiana Jaramillo-Ruiz, Irene de la Casa-Resino, Reyes Castillo-Vázquez, Pablo Ciudad-Gutiérrez, María Victoria Gil-Navarro, Ana Belén Guisado-Gil

**Affiliations:** 1 Department of Pharmacy, Virgen del Rocio University Hospital, Seville, Spain; 2 Institute of Biomedicine of Seville, Virgen del Rocio University Hospital/CSIC/University of Seville, Seville, Spain; 3 Department of Infectious Diseases, Microbiology and Parasitology, Virgen del Rocio University Hospital, Seville, Spain; 4 Centro de Investigación Biomédica en Red de Enfermedades Infecciosas (CIBERINFEC), Madrid, Spain; 5 Mass Spectrometry Laboratory/Organic Pollutants, Institute of Environmental Assessment and Water Research, IDAEA-CSIC, Barcelona, Spain; 6 Spanish National Action Plan on Antimicrobial Resistance (PRAN), Spanish Agency of Medicines and Medical Devices, Madrid, Spain

**Keywords:** anti-bacterial agents, antimicrobial resistance, antimicrobial stewardship, drug resistance, nursing homes, one health

## Abstract

**Background:**

The aging population has led to an increased number of people in nursing homes (NHs). The use of antimicrobials in this setting carries an increased risk of adverse events and interactions, particularly in individuals with advanced age, comorbidities, and polypharmacy. Antimicrobial Stewardship Programs (ASPs) have been shown to reduce the consumption of these drugs, improve prescribing practices, and have a positive ecological impact. However, the implementation of ASPs in NHs remains limited.

**Objective:**

To develop and evaluate the impact of EduFAST web platform, which is designed to train professionals, monitor activities and provide ASP teams with periodic feedback and indicators of antimicrobial use and microbial resistance in NHs.

**Methods:**

Quasi-experimental, multicenter, before-and-after study. The intervention phase will last 12 months and involves implementing ASP strategies (educational measures, indicator monitoring and periodic feedback) via the EduFAST web platform. Information will be collected on the use of the web platform, user satisfaction, knowledge gained and the number of website visits. To assess the impact of EduFAST’s implementation, the following data will be recorded: clinical variables; antimicrobial consumption and prescription profiles; and the incidence of infections caused by multidrug-resistant bacteria and *Clostridioides difficile*. Additionally, the presence and concentration of antimicrobials, multidrug-resistant microorganisms and resistance genes in wastewater from the participating centers will be recorded to evaluate the correlation between clinical samples, antimicrobial consumption and wastewater findings, as well as the environmental impact of the intervention. The intervention’s impact will be analyzed through interrupted time series analysis, trend analysis, mixed linear regression models and correlation analysis for data obtained from wastewater samples.

**Conclusion:**

The aim of this project is to facilitate the implementation and sustainability of ASPs through the development of a national-level website. Wastewater analysis will provide insight into the correlation between antimicrobial consumption, microbial resistance, and the environment from a one-health perspective.

## 1 Introduction

Due to the aging of the population, the number of people living in nursing homes (NHs) is increasing. NHs are facilities built specifically for the residential and/or nursing care of older adults with advanced physical and/or cognitive disabilities. Residents very often require personal care and support from healthcare professionals, which makes them vulnerable to easily spreadable infections (1).

In the older population, infectious diseases are one of the most important causes of mortality and have a significant impact on the increasing demand for healthcare and hospital admissions. Urinary, respiratory tract, skin and soft tissue infections are the most common infections in this setting (2, 3). Consequently, antimicrobials are among the most frequently prescribed drugs in NHs. Previous studies point out that 70% of residents receive antimicrobials, and as many as 75% of those antimicrobials were prescribed inappropriately, without adequate documentation or evidence of infection (4).

Antimicrobial Stewardship Programs (ASPs) are the strategy recommended by international organizations to improve the use of antimicrobial agents and combat bacterial resistance (5, 6).

The positive impact of ASPs in hospitals is well-established. ASPs reduce antimicrobial use and improve patient outcomes by reducing infections or colonization with multidrug-resistant (MDR) organisms, as well as reducing costs. However, evidence is still scarce in other settings, such as NHs (7–12). NHs are complex compared with hospital settings, with many factors such as the lack of on-site diagnostic resources, the availability of physicians to evaluate residents before prescribing medications or the quality of the information provided by other healthcare personnel, among others, influencing the prescribing decision-making process and, therefore, the success of ASPs (8). Among the main barriers to the implementation and maintenance of ASPs in this setting are the dispersion and heterogeneity among facilities, as well as the lack of tools for training and the periodic evaluation of process and outcome indicators (13).

On the other hand, the overuse of antimicrobials also results in pharmaceutical contamination. This refers to concerns about the impact of the release of drugs or their metabolites into the environment through urine, feces, and wastewater from urban, hospital, industrial, agricultural or livestock origin (14). Drugs can be excreted in 30–90% unchanged through urine and feces, reaching the environment after wastewater treatment, where removal is often incomplete (15). Consequently, recent studies have observed a correlation between antimicrobial consumption and resistance in wastewater (16).

The relationship between antibiotic use and antimicrobial concentrations in wastewater is garnering interest (17). Exposing bacteria to subinhibitory concentrations of antimicrobials creates the perfect environment for the development of MDR organisms and the transfer of resistance genes (18, 19). Studies conducted in hospital effluents in Japan were able to detect extended-spectrum beta-lactamase (ESBL)-producing enterobacteria, carbapenemase-producing enterobacteria, methicillin-resistant *Staphylococcus aureus* (MRSA), and multidrug-resistant *Pseudomonas aeruginosa* and *Acinetobacter baumannii* at different concentrations (20). A recently published systematic review highlighted that the prevalence of BLEE-producing enterobacteria was 24.8%, mainly *Escherichia coli* BLEE, and that the prevalence was higher in hospital effluents (21). Recent studies have highlighted the correlation between the presence of resistant microorganisms in wastewater and clinical samples (22). For example, carbapenemase genes, such as blaNDM-1 and blaOXA-23, have been identified in treated effluents and are frequently genetically connected to clinical isolates, suggesting a hazardous link between the environment and human infections (23). This has direct implications not only for ecosystems but also for wildlife and human health, as MDR organisms propagate in the environment and may eventually return to humans through the food chain or other pathways (24). On the other hand, these findings also suggest the potential use of wastewater as a non-invasive method for monitoring and surveilling microbial resistance and antimicrobial consumption in healthcare institutions and communities (25, 26).

In light of this situation, the EduFAST project intends to:

1. Design and develop a web platform to train professionals, monitor activities and provide ASP teams with regular feedback and data on antimicrobial use and microbial resistance in NHs.
2. Evaluate the impact of the web platform as an ASP tool.
3. Analyze the relationship between antimicrobial concentrations in wastewater and antimicrobial consumption at the center.
4. Analyze the relationship between the presence of MDR microorganisms and their resistance profiles in wastewater and in clinical and carrier screening samples.

## 2 Materials and methods

### 2.1 Study design and period

**Figure 1:**
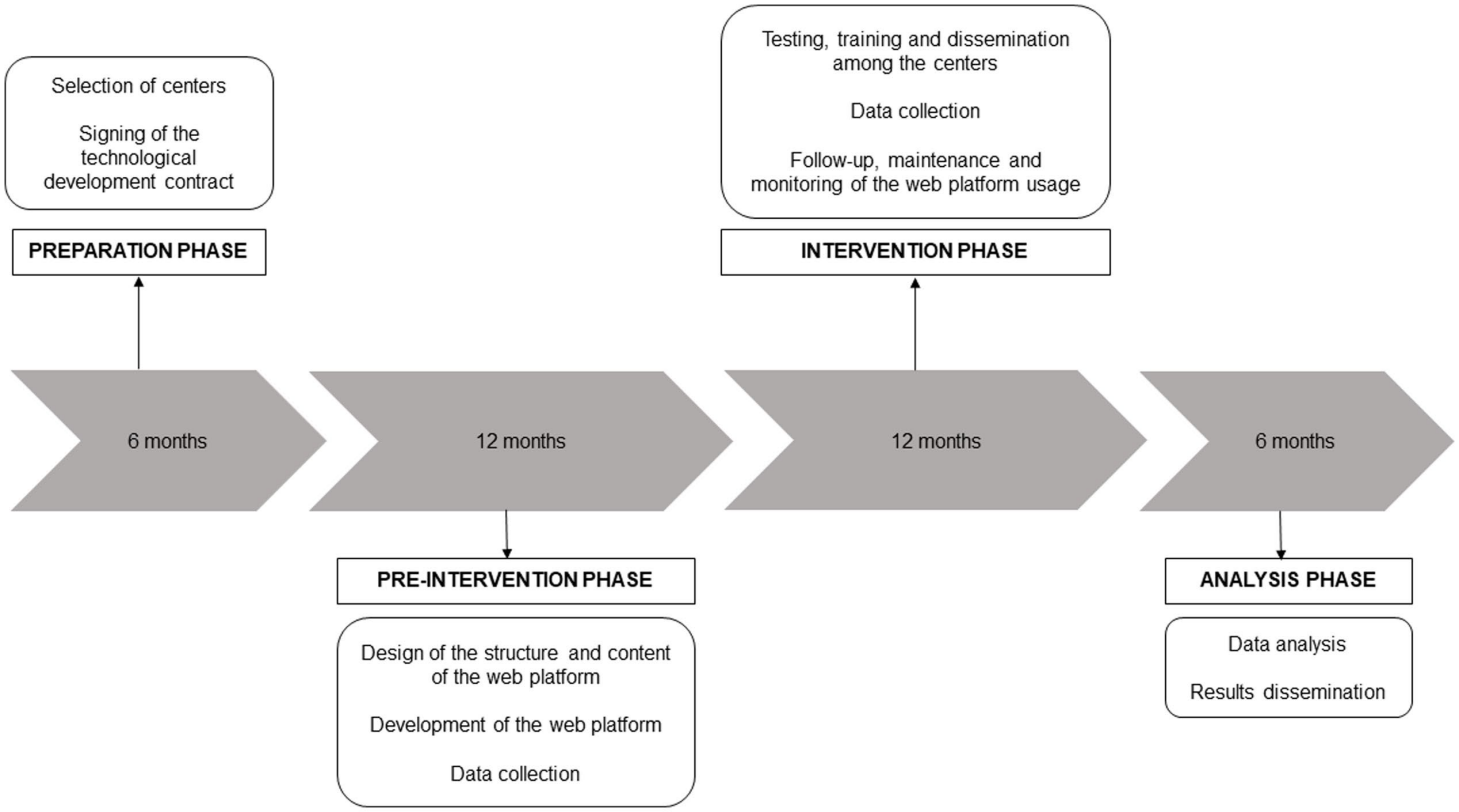
Phases and timeline of EduFAST project.

This is a quasi-experimental before-and-after interrupted time series study evaluating the implementation of antimicrobial stewardship strategies through a web platform (EduFAST) for healthcare workers in NHs. The project comprises four phases (Figure 1). The preparation and pre-intervention phase were carried out between January 2024 and June 2025. The study is currently in the intervention phase, which began in July 2025 and will continue until 2026. The results are expected in early 2027.

### 2.2 Intervention

The intervention consists of the implementation of antimicrobial stewardship strategies through the EduFAST web platform in participating NHs, to optimize antimicrobial use, improve infection management, and strengthen surveillance of antimicrobial resistance.

The intervention phase began at the end of 2025 and will continue throughout 2026. During this period, healthcare professionals from participating NHs will have access to the EduFAST platform and will be encouraged to use its different modules as part of routine clinical practice.

The intervention includes three main components:

- Educational component. Healthcare professionals will have access to training materials hosted on the platform, including treatment guidelines for the most common infectious syndromes in NHs, audiovisual materials, infographics, and simulated clinical cases. Participants will complete questionnaires assessing baseline knowledge, acquired knowledge, and satisfaction with the training activities.
- Monitoring of antimicrobial use and resistance indicators. Participating centers will enter monthly data on antimicrobial consumption, clinical outcomes, and microbiological indicators. The platform will automatically calculate predefined indicators, allowing centers to monitor antimicrobial use and resistance trends.
- Results reporting and feedback. The platform will generate periodic reports summarizing the indicators for each participating center. These reports will allow users to compare their center’s results with aggregated data from other participating NHs and will provide recommendations to improve antimicrobial prescribing practices and infection management.

### 2.3 Selection and inclusion of NHs

Study information has been distributed via email at a regional level to investigators from public Andalusian NHs who had participated in a different study carried out a few years ago (13), and at a national level through the ASP coordinators of Spain’s autonomous communities. All organizations interested in participating have received an online questionnaire to collect general characteristics of the participating NHs and, at an aggregated level, of their residents (Table 1).

**Table 1:** The selection questionnaire.

Description	Questions
General information	First and last name
	Contact email address
	Job position: doctor, pharmacist, microbiologist, nurse, nursing assistant, center director, other
	Name of nursing home
	Autonomous community
	Province
	City
Characteristics of nursing homes	Management: public, private, or mixed management
	The center serves users on a continuous basis (institutionalized), on a day basis, or both
	Number of places at the center
	Mean number of residents at the center
	The center cares for: elderly people in situations of dependency; people with physical disabilities; people with mental disabilities; people with mental health problems; people with drug addiction problems; people with chronic and/or disabling somatic illnesses; people convalescing after hospital discharge without sufficient autonomy for self-care; people with terminal illnesses; other
	Mean number of dependent residents at the center
About the center’s team of professionals	Doctor team: the center has its own medical staff available 24 h a day, every day of the week; the center has its own medical staff available less than 24 h a day, every day of the week; the center has its own medical staff available 24 h a day on working days; the center has its own medical staff available less than 24 h a day on working days; the reference medical staff belong to primary care centers; the reference medical staff belong to hospitals; other
	Nursing/Nursing assistant: the center has its own nursing/nursing assistant staff available 24 h a day; the center has its own nursing/nursing assistant staff available less than 24 h a day; other
About the prescription and dispensing of antimicrobials	When necessary, the prescription of antimicrobials is carried out through: private prescription (paper format); public prescription (paper format); electronic prescribing system (private company); electronic prescribing system (within the National Health System); no prescriptions are made at the center; other
	When an antimicrobial is prescribed, the medication is obtained from: community pharmacy; pharmacy service of the hospital of reference for the center; pharmacy service of the nursing home itself; other
	Regarding available data on antimicrobial use, we could know: total defined daily dose (DDD) of antimicrobials; DDD of specific antimicrobials; total number of antimicrobial prescriptions; number of prescriptions for specific antimicrobials; none of the above
	These data can be obtained with the following frequency: monthly; quarterly; biannual; annual; none of the above
About clinical samples for microbiological analysis	When necessary, the microbiological analysis of clinical samples is carried out at: microbiology laboratories of private hospitals; microbiology laboratories of public hospitals; other private laboratories; no samples are taken at the center for microbiological analysis; other
	Data on microbiological isolates from the residents can be obtained with following frequency: monthly; quarterly; biannual; annual; none of the above

#### 2.3.1 Inclusion and exclusion criteria

Participation in the study was possible when the NH met the following criteria:

- Public, private, or mixed management.
- Minimum center size: 50 continuous institutionalized places.
- Ability to regularly provide the required clinical data, antimicrobial consumption, and microbial resistance for the project.
- Commitment and interest in the project and achieving its objectives within the estimated timeframe.

Participation in the study was not be possible when the NH met the following criteria:

- Centers participating in another project involving the implementation of a strategy for optimizing antimicrobial use and resistance control.

#### 2.3.2 Sample size

The sample size has been calculated based on the results of the multicenter study ‘PROA-SENIOR’ (13). Considering an intraclass correlation coefficient of 0.21, a coefficient of variation between center for total antimicrobial consumption (the main variable) of 0.41, and an average of 110 residents per center, with an alpha risk of 5%, a power of 80%, and a detection of the effect size of 20%, the required sample size is 9 centers. Adding a 10% loss, the minimum sample size obtained is 10 NHs. For the wastewater analysis, three participating NHs will be selected.

### 2.4 EduFAST web platform: design and development

In terms of technological structure, the web platform will include a backend component, which will provide administration and data management features, and a web platform.

The web platform will consist of the following modules:

- *General project information module*. It will consist of two sections. The first will contain information about the research project, the web platform, and the research team. The second section will provide information about the participating NHs, with a map showing the location of each center. Access to this module will be public and available to all users.
- *Training module*. It will include treatment guidelines for the main infectious syndromes in NHs, audiovisual materials, infographics, and simulated clinical cases aimed at healthcare professionals from the participating centers. There will also be questionnaires on prior knowledge, questionnaires to assess acquired knowledge, and a satisfaction questionnaire. Access to this module will be restricted to participants, editors and administrators.
- *Data entry module*. Participants will be able to enter the numerators and denominators for each study indicator (antimicrobial use indicators, clinical indicators, and microbial resistance indicators). The web platform will then automatically calculate the final value of each indicator. Table 2 shows the indicators with their respective numerators and denominators. Access to this module will be restricted to participants, editors and administrators.
- *Results presentation and reporting module*. This module will consist of three sections. The first section, “Result Presentation,” will display the indicator results through tables and figures. This section will offer two levels of access: open access for web users, who will be able to view aggregated information on antimicrobial consumption across all participating NHs; and participant access, which will allow users to analyze their own data and compare their center’s results with the aggregated results of other centers with similar characteristics. The second section, “Result Reports,” will allow participants to download personalized reports containing their results, along with key recommendations or strategies to be implemented in the center based on deviations identified in relation to the average value of each indicator. Access to these modules will be restricted to participants, editors and administrators. The third section, “Result Maps,” will display the results of all indicators aggregated by autonomous communities on a map, and will be available only for editors and administrators.
- *Contact, help, and frequently asked questions (FAQs) module*. It will provide users with information on how to use the tool through materials that include both text and video instructions.
- *Private area*. This section will provide access to advanced features depending on the user’s role. Participants will be able to view registered users associated with their center, as well as review, modify, or delete related data. Editors/administrators will have access to features related to the maintenance, management, and generation of usage statistics for the web platform (logins, centers, users, generated reports, downloads, and interaction with materials).

**Table 2:** Indicators from the EduFAST study.

Indicator		Definition
Antimicrobial use indicators	Numerators	Monthly total Defined Daily Dose (DDD) of antimicrobials (ATC groups J01 + J02) Monthly DDD of specific antimicrobials (amoxicillin, amoxicillin/clavulanic acid, cefixime, other cephalosporins, azithromycin, clarithromycin, ciprofloxacin, levofloxacin, moxifloxacin, clindamycin, metronidazole, sulfamethoxazole/trimethoprim, fosfomycin trometamol, and other antimicrobials) Monthly number of total antimicrobial prescriptions (ATC groups J01 + J02) Monthly number of prescriptions of specific antimicrobials
	Denominator	Number of residents in the month
Clinical indicators	Numerators	Monthly number of hospital admissions Monthly number of hospital admissions due to infections Monthly number of hospital admissions due to adverse effects of antimicrobials
	Denominator	Number of residents in the month
Microbiological indicators	Numerators	Monthly number of extended-spectrum beta-lactamase-producing *Escherichia coli* isolates Monthly number of extended-spectrum beta-lactamase-producing *Klebsiella pneumoniae* isolates Monthly number of carbapenemase-producing *Enterobacteriaceae* isolates Monthly number of methicillin-resistant *Staphylococcus aureus* isolates Monthly number of multidrug-resistant *Pseudomonas aeruginosa* isolates Monthly number of multidrug-resistant *Acinetobacter baumannii* isolates Monthly detection of DNA or toxin for *Clostridioides difficile*
	Denominator	Number of residents in the month

**Figure 2:**
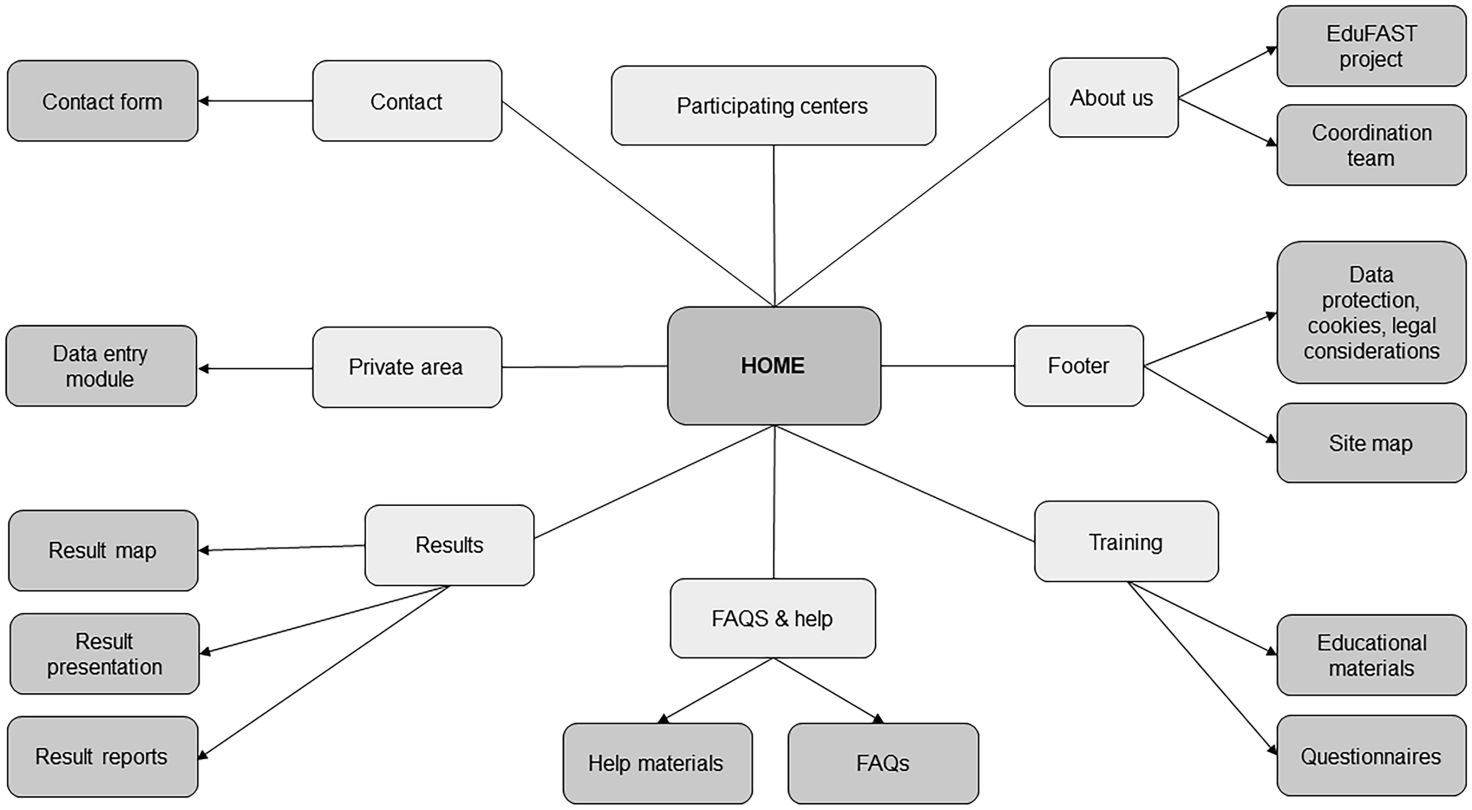
EduFAST web platform flowchart.

The flowchart corresponding to the web platform menu, which can only be accessed by participating users is shown in Figure 2.

To ensure maximum adherence, platform usage metrics will be monitored on a weekly basis and data entry on a monthly basis.

### 2.5 Data collection

The investigator team will collect data retrospectively from electronic prescription modules, NHs and microbiology unit records. During the pre-intervention phase, the data will be collected using a case report form (eCRF) in Microsoft Excel©. During the intervention phase, the data will be collected using the EduFAST web platform.

To evaluate the impact of the web platform as an ASP tool and assess the potential of using wastewater for surveillance, the following variables will be recorded monthly:

- *Variables related to the use of the web platform*: number of visits to the web platform, number of NHs registered, activity on the web platform (reports, completed training, etc.), and the average score on knowledge and satisfaction questionnaires.
- *Variables of antimicrobial use*: Defined Daily Dose (DDD) according to the World Health Organization (27), globally (Anatomical Therapeutic Chemical (ATC) groups J01 + J02), and for specific antimicrobials (amoxicillin, amoxicillin/clavulanic acid, cefixime, other cephalosporins, azithromycin, clarithromycin, ciprofloxacin, levofloxacin, moxifloxacin, clindamycin, metronidazole, sulfamethoxazole/trimethoprim, and fosfomycin trometamol). We will also record the number of prescriptions for the aforementioned antimicrobials, both individually and globally.
- *Clinical variables*: number of residents, number of hospital admissions, number of hospital admissions due to infections, and number of hospital admissions due to adverse effects of antimicrobials.
- *Microbiological variables*: number of isolations of MDR microorganisms (ESBL-producing *E. coli*, ESBL-producing *Klebsiella pneumoniae*, carbapenemase-producing *Enterobacteriaceae*, MRSA, MDR *P. aeruginosa*, and MDR *A. baumannii*) in clinical samples and also in nasal and rectal or perineal swab samples (only in selected NHs); and number of DNA or toxin detection for *C. difficile* in clinical samples.
- *Environmental variables (only in selected NHs)*: concentration of antimicrobials and their metabolites (amoxicillin, methylated amoxicillin, (5R,5S)-amoxicillin, clavulanic acid, cefixime, azithromycin, clarithromycin, levofloxacin, ciprofloxacin, moxifloxacin, clindamycin, clindamycin sulfoxide, metronidazole, hydroxy-metronidazole, sulfamethoxazole and trimethoprim), and concentration of MDR microorganisms (ESBL-producing *E. coli*, ESBL-producing *K. pneumoniae*, carbapenemase-producing *Enterobacteriaceae*, MRSA, MDR *P. aeruginosa*, MDR *A. baumannii*) in wastewater samples.

### 2.6 Sample collection

#### 2.6.1 Human samples

Clinical samples, such as urine, sputum cultures, percutaneous aspirations, and exudates, will be collected during routine clinical care when clinically indicated through the pre-intervention and intervention phases and analyzed in the laboratory of each participating center. To identify carriers of MDR bacteria (carrier screening samples), nasal swabs will be collected to detect MRSA and rectal or perianal swabs to detect ESBL- and carbapenemase-producing *Enterobacterales*, MDR *P. aeruginosa* and *A. baumannii*, from residents in centers participating in wastewater analysis. These screening samples will be collected quarterly by nurses from residents who have provided written informed consent, during the intervention phase, between the first and the last day of this phase. These samples will be collected using swabs, sent in a bacterial transport medium, and processed in the same way as clinical samples for the phenotypic study in the coordinator center’s laboratory.

#### 2.6.2 Wastewater samples

For the analysis of wastewater in the selected NHs, a sample of between 500 and 1,000 mL will be collected from the drainage manhole of each center once a month between 8:00 and 10:00 a.m. A total of at least 24 samples will be collected throughout the study (12 during the pre-intervention phase and 12 during the intervention phase). If a center has more than one drainage manhole, samples will be collected from all of them. These samples will be transported under refrigeration to the coordinating center. There, four 50 mL aliquots will be prepared: two will be used to determine the levels of antimicrobials and their metabolites, and the other two for the analysis of microorganisms and resistance genes.

### 2.7 Microbiological analysis

#### 2.7.1 Human samples

For phenotypic characterization, selective culture media will be used for the recovery of MDR microorganisms (Thermo Scientific™ Brilliance™ ESBL/Brilliance CRE biplate). The media will be incubated for 48 h, with a first reading at 24 h, under aerobic conditions at 35–37 °C. The MicroScan Walkaway semi-automated microdilution system (Beckman Coulter Inc., USA) will be used for the identification and susceptibility study. The analysis of antibiotic susceptibility and resistance mechanisms will be performed according to the criteria of the European Committee on Antimicrobial Susceptibility Testing (EUCAST) (28).

The following criteria will be applied to the MDR classification described in Table 3.

**Table 3:** Definitions of MDR classification.

Multidrug-resistant organisms	Definitions
Carbapenemase isolate	If the isolate produce both ESBL and carbapenemase
ESBL-producing isolate	If the isolate is resistant to meropenem, imipenem, and/or ertapenem but does not produce carbapenemases and produces ESBL
MDR *P. aeruginosa*	If the isolate is resistant to carbapenems or ≥3 classes of antipseudomonal agents (penicillins [piperacillin-tazobactam], cephalosporins, carbapenems, quinolones, or aminoglycosides)
MDR *A. baumannii*	If the isolate is resistant to carbapenems or ≥3 classes of antibiotics (penicillins [piperacillin-tazobactam], cephalosporins, carbapenems, quinolones, or aminoglycosides)

#### 2.7.2 Wastewater samples

Active surveillance will be carried out for the presence of MDR microorganisms in the wastewater from selected NHs. Simultaneously, phenotypic and molecular characterization will be performed.

From each wastewater sample, 50 mL aliquots will be obtained and centrifuged at 12,000 rpm for 10 min. From the bacterial pellet obtained, an amount equivalent to 5 μL will be taken and resuspended in 500 μL of nucleic acid-free water. Then, 10 μL will be taken and quantitatively plated on Columbia blood agar. For the detection of MDR bacteria, 10 μL will be taken and planted in selective media. Then, the same process described for clinical samples will be performed for phenotypic characterization. For molecular characterization, the MDR Direct Flow Chip® system (Vitro S.A., Granada, Spain) (29) will be used, based on a polymerase chain reaction (PCR) and reverse hybridization system that allows the detection of five bacterial species (*S. aureus, P. aeruginosa, E. coli, K. pneumoniae* and *A. baumannii*), as well as 56 genetic determinants of antibiotic resistance.

### 2.8 Antimicrobial concentration in wastewater samples

First, the sample will be centrifuged 20 min at 3,000 rpm and at a temperature below 15 °C. Then, it will be divided into two aliquots and adjusted to pH 2 or 7 for extraction of the following antimicrobials:

- pH 2: amoxicillin, methylated amoxicillin, amoxicillin (5R,5S), clavulanic acid, cefixime, clindamycin, clindamycin sulfoxide, levofloxacin, ciprofloxacin, and moxifloxacin will be extracted. The last three will also be extracted at pH 7.
- pH 7: azithromycin, clarithromycin, metronidazole, hydroxy-metronidazole, sulfamethoxazole, and trimethoprim will be extracted.

Compound extraction will be performed using solid-phase extraction (SPE) with Oasis HLB cartridges (6 cc, 200 mg; Waters, Labtim, Croatia). Finally, the extract will be dried under a stream of nitrogen and reconstituted with the mobile phase. The resulting extract will be refrigerated and transported to the Institute of Environmental Assessment and Water Research of the Spanish National Research Council (IDAEA-CSIC).

There, the analysis and quantification of antimicrobials and metabolites will be carried out by means of an Orbitrap-Exactive HCD (Thermo Fisher Scientific, Bremen, Germany) mass spectrometer equipped with a heated electrospray source (H-ESI II), a Surveyor MS Plus pump and an Accela Open AS autosampler which will be kept at 10 °C (Thermo Fisher Scientific, San Jose, California). The chromatographic separation will be performed on a reversed-phase Luna C18 column (150 × 2.0 mm, 5 μm, Phenomenex, Torrance, CA, USA) preceded by a C18 guard column (4 × 2.1 mm, Waters, Milford, MA, USA). The mobile phase will be composed of 0.1% HCOOH in water as solvent A and 0.1% HCOOH in ACN as solvent B at a flow rate of 200 μL min^−1^. The linear gradient elution program will be: 5% up to 95% ACN in 15 min. This percentage will then be maintained for 1 min, and after that the LC system will return to initial conditions for 10 min. The injection volume will be 10 μL. The MS analyses will be carried out in positive and negative electrospray ionization (ESI). Data will be acquired in full-scan mode in a mass range of *m/z* 100–1,000. A maximum of ±5 ppm mass accuracy extraction window will be applied for peak identification. The analytes will be quantified using TraceFinder 5.1 software (Thermo Fisher Scientific, Bremen, Germany).

**Figure 3:**
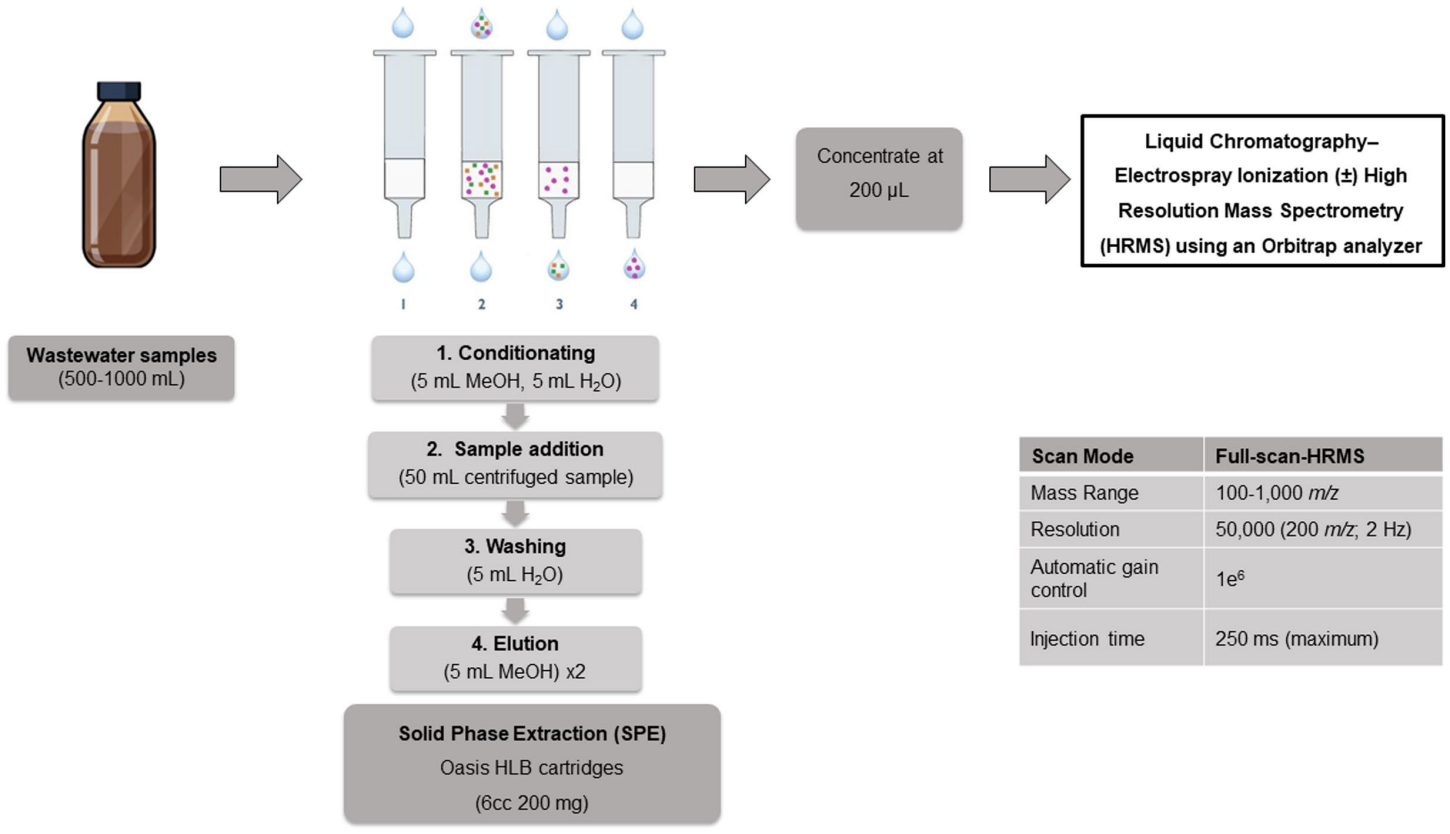
Analytical methodology for analysis of antimicrobials in wastewater samples.

The analytical methodology for analysis of antimicrobials in wastewater samples is shown in Figure 3.

### 2.9 Statistical analysis

A descriptive analysis will be performed. Continuous variables will be expressed as measures of central tendency and dispersion, such as mean ± standard deviation or median and interquartile range, depending on the distribution. Categorical variables will be expressed as absolute and relative frequencies.

Interrupted Time Series Analysis (ITSA) will quantify changes in the level (immediate effect) and trend (sustained effect) of outcomes associated with implementing the web platform, considering the study’s longitudinal and ecological nature. ITSA will be performed using a segmented regression model to estimate four parameters: baseline level, baseline trend, the change in level immediately following the intervention, and the change in trend following the intervention. Seasonal adjustment will be applied to those time series in which seasonality were detected. The model incorporates an autoregressive term to account for the autocorrelation present in the time series data. To assess variability and the overall effect across different centers, we will use a linear mixed model (LMM) for repeated measures that includes a random intercept for center. Potential confounders will be managed as fixed effects covariates. *Post hoc* comparisons of estimated marginal means will be performed with the Bonferroni correction applied to adjust for multiple comparisons.

We will evaluate the correlations between the presence of MDR microorganisms and their resistance profiles in wastewater, clinical, and carrier screening samples. We will also evaluate the correlations between antimicrobial concentrations in wastewater and consumption data from centers. We will assess the strength and direction of these correlations using the appropriate Pearson or Spearman correlation coefficient. Then, we will perform univariate and multivariable linear regression analyses to further explore the relationships.

All statistical analyses will be performed using two-tailed tests in IBM SPSS Statistics and R statistical software. A *p*-value of less than 0.05 will be considered statistically significant.

## 3 Discussion

The success of ASPs hinges on continuing education and motivating healthcare professionals, as well as periodically measuring specific indicators related to clinical outcomes, antimicrobial use, and microbial resistance. Currently, these tools are lacking in NHs, which hinders the training of professionals and the monitoring of these indicators (30). Against this backdrop, EduFAST is being developed to address this need by providing a web platform that will facilitate the training of professionals and the monitoring of key parameters for ASP teams. The development and implementation of EduFAST will contribute to the implementation of antimicrobial stewardship strategies and ultimately improve the health of NH residents.

The improvements in antimicrobial use, reductions in the incidence of infections caused by MDR microorganisms, and decreases in associated healthcare costs led by ASPs in hospitals and primary care supports the need to implement these programs in NHs (31, 32). For this reason, the Centers for Disease Control and Prevention have recommended the establishment of ASPs in these centers, backed by a set of general measures (6). In the Spanish context, the Spanish National Action Plan on Antimicrobial Resistance (PRAN) has established a specific line of work to design the framework and strategy for the implementation of ASPs in NHs (33).

A previous systematic review and meta-analysis conducted by the project’s research team concluded that ASPs could reduce antimicrobial consumption in this setting without negatively impacting hospital admissions or mortality. However, it should be noted that few studies met the necessary quality standards to draw definitive conclusions on this matter. All of the studies included in the review used clinical practice guidelines and educational strategies as ASP tools (8). However, only two of the studies applied information and communication technology (ICT) strategies (34, 35). This contrasts with the expert recommendation guidance document for ASPs published in 2023, which states that e-tools should be included in ASP interventions whenever possible and feasible to facilitate guidelines, clinical pathways, and post-prescription reviews. The efficacy and safety of ASP e-tools should be validated through cluster randomized control trials or adequately controlled quasi-experimental designs, and ASP e-tools should be available to prescribers (36). The EduFAST project has been designed to meet these recommendations and bridge the gap in ICT use in ASPs in NHs.

Among ASP studies that employed ICT strategies, there were webinars, posters, and pocket cards aimed at healthcare professionals, such as nurses, pharmacists, and prescribers, to reduce the unnecessary use of antimicrobials for uncomplicated cystitis among non-catheterized residents (34, 35). Similarly, the EduFAST project will design a free web platform for healthcare professionals in NHs. However, EduFAST will include innovative features such as text documents containing information from therapeutic guidelines and clinical cases presented in video format. Additionally, questionnaires will be administered to assess participants’ knowledge before and after viewing the educational materials. The educational material within the EduFAST project will encompasses ASP principles, preventive measures of infection, and the diagnosis and treatment of main infectious syndromes, with a particular emphasis on the most prevalent ones (urinary tract, respiratory tract, and skin and soft tissue infections). However, this is a distinct point because most studies have focused exclusively on urinary tract infections (34, 37, 38) and, to a lesser extent, respiratory tract infections (39).

To evaluate the impact of ASPs, previous studies have analyzed changes in the number of prescriptions or, less frequently, in the DDD as an internationally accepted measure of antibiotic use (8). Indicators of antimicrobial resistance are infrequently reported, and the outcomes vary among studies, although common outcomes include *C. difficile* detection, MRSA and/or MDR gram negative bacterial infections (13, 34, 40, 41). Similarly, clinical outcomes (e.g., hospitalization rates, mortality, and clinical adverse events) are reported in fewer than one-third of ASP studies in NHs, according to a recent systematic review (41). The EduFAST project will collect antimicrobial consumption, resistance, and clinical indicators, allowing for comparison with the results of previous interventions. But also, EduFAST project will aim to evaluate the environmental impact by studying the concentration of antimicrobials, MDR microorganisms, and resistance genes in wastewater. Currently, there is little evidence regarding the impact of antibiotic use on the microbiological composition of wastewater in NHs and the potential use of wastewater for surveillance purposes of antimicrobial consumption and microbial resistance (42, 43). This project will therefore provide relevant information on this subject and contribute an environmental indicator that has not been included in ASPs previously.

The primary limitation of the study is its quasi-experimental, before-and-after design. However, given the recommendations of national and international organizations to promote the implementation of ASP in all healthcare settings and the existing evidence of its positive impact, proposing a clinical trial with a non-intervention comparison group would raise significant ethical concerns. Another limitation is the heterogeneity among NHs, particularly with regard to size, resident profiles, infrastructure, and human resource availability. This could lead to variability in implementation and affect adherence to the recorded variables. For this reason, we have implemented a system of close monitoring and follow-up of platform usage metrics and indicators, as described in the Materials and Methods section. Second, linear mixed models will be used to evaluate the intervention’s impact and handle center effects in this multicenter study.

In conclusion, implementing this project involves developing an interactive web platform to facilitate professional training and enable monitoring of indicators related to antimicrobial resistance and use in NHs. This initiative is expected to generate significant health benefits, including reducing overall antimicrobial consumption in these settings, optimizing prescribing patterns by further limiting the use of broad-spectrum antibiotics, reducing infections caused by MDR bacteria and *C. difficile*, and facilitating ASP interventions in NHs. Wastewater analysis will provide insight into the correlation between antimicrobial consumption, microbial resistance, and the environment, exploring its potential use for surveillance.
